# Nucleofection of Rat Pheochromocytoma PC-12 Cells with Human Mutated Beta-Amyloid Precursor Protein Gene (*APP-sw*) Leads to Reduced Viability, Autophagy-Like Process, and Increased Expression and Secretion of Beta Amyloid

**DOI:** 10.1155/2015/746092

**Published:** 2015-03-10

**Authors:** Beata Pająk, Elżbieta Kania, Arkadiusz Orzechowski

**Affiliations:** ^1^Electron Microscopy Platform, Mossakowski Medical Research Centre, Polish Academy of Sciences, Pawińskiego 5, 02-106 Warsaw, Poland; ^2^Department of Physiological Sciences, Faculty of Veterinary Medicine, Warsaw University of Life Sciences (SGGW), Nowoursynowska 159, 02-776 Warsaw, Poland

## Abstract

Pheochromocytoma PC-12 cells are immune to physiological stimuli directed to evoke programmed cell death. Besides, metabolic inhibitors are incapable of sensitizing PC-12 cells to extrinsic or intrinsic apoptosis unless they are used in toxic concentrations. Surprisingly, these cells become receptive to cell deletion after human *APP-sw* gene expression. We observed reduced cell viability in *GFP* vector + *APP-sw*-nucleofected cells (drop by 36%) but not in *GFP* vector − or *GFP* vector + *APP-wt*-nucleofected cells. Lower viability was accompanied by higher expression of A*β* 1-16 and elevated secretion of A*β* 1-40 (in average 53.58 pg/mL). At the ultrastructural level autophagy-like process was demonstrated to occur in *APP-sw*-nucleofected cells with numerous autophagosomes and multivesicular bodies but without autolysosomes. Human *APP-sw* gene is harmful to PC-12 cells and cells are additionally driven to incomplete autophagy-like process. When stimulated by TRAIL or nystatin, CLU protein expression accompanies early phase of autophagy.

## 1. Introduction

Pheochromocytoma (PCC) is a rare neuroendocrine tumor located in adrenal medulla which secretes massive quantities of catecholamines with malignant hypertension as the fatal outcome. The treatment of choice is surgery associated with high-risk complications (refractory hypertension). In laboratory conditions, pheochromocytoma cells undergo differentiation to neural cells upon treatment with nerve growth factor (NGF) [1]. Alternatively, pheochromocytoma cells could be manipulated with selected gene insertions/silencing leading to additional phenotypic modifications (neural) that cease disproportionate endocrine activity. Accordingly, rat pheochromocytoma cells (PC-12 cell line) are frequently used in* in vitro* studies as a cellular model of neurodegenerative diseases. Alzheimer's disease (AD) is the most prevalent neurodegenerative disease. The hallmark is the extracellular deposition beta amyloid (A*β*) accompanied by dementia and progressive loss of cognitive processes. Familial form (early onset associated with mutations in beta-amyloid precursor protein gene,* APP*) is rare, whereas sporadic form prevails (late onset, 95% of cases). Notably, the molecular mechanisms of neuronal decay in hippocampus and prefrontal cortex in AD remain ambiguous despite extensive studies carried out for more than a century [2]. Formation of A*β* is associated with the activity of membrane bound *γ*-secretase complex including catalytic role of presenilins (PS-1 and PS-2). This complex attacks remaining of APP just after proteolytic *α*-secretase (nonfibrillogenic) or *β*-secretase (fibrillogenic) processing. Shorter isoforms of APP (695 amino acids and less) are specific substrates for *α*-secretase (ADAM10) while others (751 amino acids and more) are specific for *β*-secretase (BACE1). Protein products of mutated* APP* gene (amino acid substitutions) are main targets of BACE1 regardless of APP length [3]. Key players in A*β* formation are located in lipid rafts (LR), nanodomains formed by deposition of cholesterol in membrane lipid bilayers [4, 5]. Lipid rafts are cognate platforms for several signaling pathways including death receptor ligands (DRL) widely known in etiology of AD [6]. Brain is unique in both highly self-sufficient cholesterol (CHOL) metabolism, macroautophagy (autophagy), and DRL (TNF-*α*, TRAIL) secretion by microglia [7] as either process is independent from peripheral regulation. Thus, autophagy in neurons is constitutive rather than cellular adaptation to nutritional stress as brain is specially protected tissue with constant supply of nutrients even under starvation [8]. Aging is known to differently affect brain CHOL metabolism, autophagy, and DRL activity. Age-dependent onset of neurodegenerative diseases most likely correlates with age-dependent increase of brain cholesterol and DRL secretion [9] as well as age-dependent decline of autophagic activity. The former is associated with altered CHOL metabolism and the latter with unknown changes in control of autophagy and DRL [10]. With the progress of aging, the average concentrations of DRL become elevated [11]. Clusterin protein (CLU) is the rare extracellular ATP-independent chaperone present in both body fluids and tissues where it acts either as cytoprotective (glycosylated-secreted form) or cytotoxic (unglycosylated-intracellular form) factor [12–15]. CLU is upregulated in the brains of individuals affected by neurodegenerative diseases [16, 17], via most likely outcome of ER stress-induced calcium depletion and debilitated N-glycosylation resulted in intracellular accumulation of N-glycan deficient proteins [18]. Although neurodegeneration accompanying cell death mechanism (apoptosis, necrosis, necroptosis (aponecrosis), paraptosis (cell death type 3), or autophagic cell death) is still a matter of debate, ER stress and unfolded protein response (UPR) are frequently associated with impaired autophagy [19, 20].

Autophagy starts when isolation double membrane bilayer (phagophore probably starts off in endoplasmic reticulum) appears in the cytosol, where it engulfs cytoplasmic constituents including organelles. Elongation of the isolation membrane around the region of cytoplasm and closure of the inner and outer bilayers of the isolation membrane leads to formation of double membrane autophagosome. To digest the contents, autophagosome must fuse with lysosome to create autolysosome. Before fusion with lysosome, it may also mingle with endosome to form amphisome. In neurons, autophagosomes are located in the cytoplasm but lysosomes are restricted mainly to juxtanuclear region. Apparently, autophagosomes produced in dendrites or neurites have to be transported to the lysosomes in the cell body by retrograde movement along microtubules. Young neurons achieve this task relatively easily but it is not the case for old neurons. Without efficient autophagy, neurons accumulate ubiquitinated protein aggregates and degenerate [20]. Autophagy can be monitored by targeting proteins present in the outer membrane of autophagic vacuoles. MAP LC3 (microtubule-associated protein 1 light chain 3/MAP1LC3) a small subunit of MAP-1A/MAP-1B is subject of subsequent processing by Atg4B protease while the processed form (LC3-I) is further modified by Atg7 and Atg3 to become coupled with phosphatidylethanolamine (PE), an important component of inner leaflet of lipid rafts. Thus, the PE-conjugated form, designated as LC3-II, is available for embedment with outer and inner membrane of autophagosome [21]. Initiation step in autophagy (selection and sequestration) is the translocation of Beclin 1 (mammalian ortholog of yeast Atg6) and VPS34 assembled in multiprotein complex to preautophagosomal structures (PAS) [22]. Once substrates (proteins and/or organelles) for autophagy are bound to specific receptors (p62, NBR1, NDP52, optineurin (OPTN), histone deacetylase 6 (HDAC6), or NIX) they can join LC3-II and Atg12-Atg5 to become core of future autophagosome [23]. Phagophore elongates with subsequent closure which permits isolation of the cargo trapped in autophagic vacuole surrounded by double membrane [24]. The last step, process of substrate digestion, requires lysosome degradative machinery, and hence autophagosomes fuse with lysosome for the execution phase of autophagy. The principal role of autophagy is to accomplish cellular needs to cope with various stressors such as deficit of essential amino acids or ATP or both, lack of growth factor signals, oxygen debt, build-up of damaged proteins, or endoplasmic reticulum stress [25]. The fundamental task in cellular homeostasis is played by lysosomes. Whatsoever, macroautophagy, mitophagy, or chaperone-mediated autophagy the final common path is lysosomal digestion of sequestered substrates. Obviously, lysosome is the target and stand organelle for primary regulator (mTOR complex I, TORC1) which antagonizes release of amino acids. When TORC1 becomes inactive, the lysosomes encourage autophagy through control of substrate sequestration. Although regulatory network of autophagy is complex and far from complete, the outline of basic processes at the molecular level is generally well known.

In this experiment, we hypothesized that mutated* APP* gene (*APP-sw*) might be helpful in making pheochromocytoma cells less viable and more susceptible to cell death triggering mechanisms. Foundations of assumption were based on results acquired from former study pointing to higher mortality of PC-12 cells transfected with the mutated* APP* gene [26–28]. Nevertheless, up to date the research was forced to study apoptosis and/or necrosis. Our preliminary observations with transmission electron microscopy revealed that PC-12 cells nucleofected with mutated* APP-sw* gene show symptoms of extensive autophagy-like process which is frequently observed in neurodegenerative diseases. The novelty of this study is that it put concern on the process of autophagy widely believed to be survival mechanism, even though its anomalous course leads to cell deletion (cell death type 2). As APP processing is often located in lipid rafts reliant on cholesterol, we tested statins and cholesterol chelator M*β*CD. Additionally, pheochromocytoma cells are immune to death receptor ligands [29] so alternate use of TNF-*α* and TRAIL was justified with regard to cell viability, APP processing, and molecular markers of autophagy. Intracellular CLU expression was monitored to check if the protein is accumulated in transgene-bearing cells.

Overall, this study should shed more light on the molecular mechanisms of cell death related to tumor cell differentiation and changes observed in cellular models of neurodegenerative diseases.

## 2. Materials and Methods

### 2.1. Materials

Media (Dulbecco's modified Eagle's medium (DMEM) low glucose (5.5 mM), F-12K medium, Kaighn's Modification of Ham's F-12 medium with Glutamax), PBS (including Ca^2+^ and Mg^2+^), antibiotics, heat inactivated sera (fetal bovine serum (FBS) and horse serum (HS)) were purchased from Gibco Life Technologies (Grand Island, NY, USA). Nerve growth factor (NGF), tumor necrosis factor alpha (TNF-*α*), and tumor necrosis factor alpha-related apoptosis inducing ligand (TRAIL) (Sigma Aldrich Chemical Co., St. Louis, MO, USA) were dissolved according to manufacturer. Metabolic inhibitors (atorvastatin: ATOR, simvastatin: SIM, nystatin: NY, methyl-beta-cyclodextrin: M*β*CD, rapamycin: RAPA, and chloroquine: CHLOR) if necessary were dissolved in DMSO. All other reagents were cell culture tested, of high purity, and unless otherwise stated they were purchased from Sigma-Aldrich Chemical Co. (St. Louis, MO, USA). Plastics, including BioCoat Collagen IV coated Cellware, were from Becton Dickinson (BD Biosciences, Franklin Lakes, NJ, USA) and tubes for deep freezing from Nunclon (Nunc, Roskilde, Denmark) while syringe filters were purchased from Corning-Costar Inc. (Cambridge, MA, USA).

### 2.2. Rat Pheochromocytoma PC-12 Cell Cultures and Treatments

The rat PC-12 cell line was obtained from European Collection of Animal Cell Cultures (ECAAC). Cells were initially suspended in growth media (GM) containing DMEM with Glutamax supplemented with 10% (v/v) fetal bovine serum (FBS) + 5% (v/v) horse serum (HS), pen : strep (penicillin : streptomycin solution, 50 IU/mL/50 *μ*g/mL), gentamicin sulfate 20 *μ*g/mL, and Fungizone (amphotericin B, 1 *μ*g/mL) and plated onto plastic noncoated culture flasks. G-418 (400 *μ*g/mL) and ampicillin (100 *μ*g/mL) were additionally present in GM for growth and selection of positively transfected PC-12 cells. They were cultured at 37°C in a humidified 5% CO_2_ and 95% air in incubator. After reaching 70–80% confluence, PC-12 cell suspensions were subcultured, and the same quantity of cells was seeded onto 60 mm Petri dishes, 96-multiwell plates (Becton Dickinson, BD Biosciences, Franklin Lakes, NJ, USA) depending on the experimental protocol. For differentiating and differentiation states when PC-12 cells reached 80% confluence, growth medium was switched to differentiation medium (DM) containing DMEM with Glutamax supplemented with 4% (v/v) fetal bovine serum (FBS) + 2% (v/v) horse serum (HS) + NGF (50 ng/mL) and the same antibiotic : antimycotic mixture. Cells were allowed to differentiate for 48 h and the DM was replaced by freshly prepared serum-free reference medium (RM) containing 0.1% BSA (w/v) + NGF (50 ng/mL) with or without experimental factors for another 48 h (48 h/48 h). When the experimental factors were dissolved in DMSO, the equivalent volume of vehicle (0.1% v/v) was added to the control cells. If necessary the same quantity of differentiated cells with neural phenotype was removed from the culture plates using 0.5% (w/v) trypsin-EDTA (harvesting), centrifuged in DM at 200 ×g for 5 min, media were aspirated, and cell pellets were resuspended in RM. Media were changed every other day. Cell monolayers were harvested for Western blots or PCR. Floating dead cells were removed during media change or washed with PBS and were not included in these experiments.

### 2.3. Cloning and Expression Vectors of the* Homo sapiens* Beta-Amyloid Precursor Protein Gene 1-695 (Transcript Variant 3,* APP-wt*), Swedish Mutation in* Homo sapiens* Beta-Amyloid Precursor Protein Gene* APP-KM670/671NL* Double Mutation in the APP Gene Resulting in Amino Acid Substitutions of Lys to Asn (Codon 670) and Met to Leu (671), and PrecisionShuttle Mammalian Vector with C-Terminal Tag GFP (*pCMV6-ACGFP*)

Cloning and expression of the* Homo sapiens* beta-amyloid precursor protein gene 1-695 (transcript variant 3,* APP-wt*), Swedish mutation in* Homo sapiens* A*β* precursor protein gene* APP-KM670/671NL* double mutation in the* APP* gene resulting in amino acid substitutions of Lys to Asn (codon 670) and Met to Leu (671), and PrecisionShuttle mammalian vector with C-terminal tag GFP (*pCMV6-ACGFP*) carrying resistance genes to antibiotics (*Neo* ^*r*^ and* Amp* ^*r*^) were ordered in OriGene Technologies, Inc., Rockville, MD, USA, and purchased from STI Bartosz Czajkowski (Poznań, Poland). Both genes (*APP-wt* and* APP-sw*) were expressed under* GFP* promoter (Supplementary material Figure 1 available online at http://dx.doi.org/10.1155/2014/746092). Mock-nucleofected cells (reference, “M”) underwent complete procedure except for no vector was added prior to nucleofection.

### 2.4. Transfection of PC-12 Cells with the* Homo sapiens* Beta-Amyloid Precursor Protein Gene 1-695 (Transcript Variant 3,* APP-wt*), Swedish Mutation in* Homo sapiens* Beta-Amyloid Precursor Protein Gene* APP-KM670/671NL* Double Mutation in the APP Gene Resulting in Amino Acid Substitutions of Lys to Asn (Codon 670) and Met to Leu (671), and PrecisionShuttle Mammalian Vector with C-Terminal Tag GFP (*pCMV6-ACGFP*)

The procedure for PC-12 cell transfection was based on the method described by the manufacturer (Amaxa Cell Line Nucleofector Kit V, Lonza Cologne AG, Cologne, Germany). Program was used for Nucleofector 4D. In short, 6-multiwell plates were prepared by filling appropriate number of wells with 1.4 mL of media (Ham's F-12 with 15% HS + 2.5% FCS with Glutamax, 1.5 g/L sodium bicarbonate without antibiotics). Plates were preincubated/equilibrated in a humidified 37°C/5% CO_2_ incubator. To obtain single cell suspension the PC-12 clusters were passed through a 22-gauge needle (10–15 times). The required number of cells (2 × 10^6^ cells per sample) was centrifuged in sterile Eppendorf tubes (1.5 mL) of molecular purity at 200 ×g for 10 minutes at room temperature. Supernatant was removed completely, and cell pellets were resuspended carefully in 100 *μ*L room-temperature complete nucleofector solution. An aliquot of 100 *μ*L of cell suspension was mixed with 2 *μ*g DNA of appropriate vector. Then, cell/DNA suspension was transferred into certified cuvette and closed with a cap and after selection the appropriate program nucleofection was performed. The cuvettes were left for 15 min in room temperature; afterwards 500 *μ*L of the preequilibrated culture medium was added to the cuvette and samples were gently transferred into the prepared 6-well plate (final volume 1.9 mL media per well). Cells were analyzed 24 hours after nucleofection using light (A) and fluorescence microscopy (B) (supplementary material Figure 2). Average transfection efficiency of PC-12 cells after 24 hours after nucleofection was analyzed by flow cytometry. Viability was measured by using MTT assay. One day after nucleofection, cells were used to carry out the experiments.

### 2.5. Determination of Cell Viability

Cell viability was based on the ability of cells grown on 96-multiwell plates to convert soluble MTT [3-(4,5-dimethylthiazol-2-yl)-2-5-diphenyltetrazolium bromide] into an insoluble purple formazan reaction product with minor modifications to protocol described [30]. Briefly, cells were uniformly seeded in 96-multiwell flat bottomed plates and grown in GM. Confluent cultures were washed with PBS and then exposed to DM for 48 h. Afterwards, DM was replaced by RM with or without experimental factors for another 48 h. Relative viability (percentages of mean control value) was evaluated. To do this, media were removed and cells were washed with PBS and were further incubated with MTT for 1 h at 37°C in a humidified 5% CO_2_ and 95% air in incubator. Next, MTT solution was removed and water insoluble formazan was immediately dissolved in DMSO. Alternatively, cell viability was determined on the basis of modified lysosomal uptake of neutral red dye [31]. Viable cells will take up the dye by active transport and incorporate the dye into lysosomes, whereas nonviable cells will not take up the dye. PC-12 cells were grown in 96-multiwell flat-bottomed plates in GM. After reaching confluence, cells were switched into postmitotic status by incubation in DM (differentiation) for 48 h. Next, wells were immersed with RM (CTRL) and experimental media for another 48 h (percentages of control value). For the additional 1 h of incubation, these media were replaced by 50 *μ*L neutral red (NR) reagent (5 mg/mL in PBS, sterilized by filtration). After incubation, the medium was aspirated and cells were washed with PBS. Cell monolayers were allowed to dry at ambient temperature, and neutral red accumulated within lysosomes of living cells was dissolved by addition of 100 *μ*L DMSO (70% in H_2_O).

The absorbances for MTT and neutral red were measured at 490 and 550 nm, respectively, with ELISA reader type Infinite 1000 (TECAN, Austria). Relative percentages (versus nontreated controls) of viable cells were measured by MTT conversion into purple formazan and accumulation of neutral red in intact lysosomes, respectively.

### 2.6. Determination of Apoptosis and Necrosis

To evaluate apoptosis (percent YO-PRO-1 positive cells) and necrosis (percent propidium iodide positive cells) the cells were seeded at black 96-well multiplates with transparent bottom (BD Biosciences, Franklin Lakes, NJ, USA). Confluent cultures were washed with PBS and then exposed to DM for 48 h. Afterwards, DM was replaced by RM with or without experimental factors for another 48 h. Relative percentages of apoptosis and necrosis (versus nontreated controls) were measured according to the method adapted from Plantin-Carrenard et al. [32]. To do this, media were removed, cells were washed with PBS and were further incubated with YO-PRO-1 (1 *μ*M), and propidium iodide (10 *μ*g/mL, PI) dissolved in PBS for 0.5 h at 4°C on ice in the dark. After incubation, YO-PRO-1 and PI accumulated in apoptotic and necrotic cells, respectively. The validity of the method was verified by observations in fluorescent microscope (Olympus IX71 Series, Osaka, Japan). For YO-PRO-1, the fluorescence was measured using the optimum wavelengths of 485 nm (*λ*_ex_) and 530 nm (*λ*_em_). Simultaneously, the fluorescence of PI bound to nucleic acids was measured using the optimum wavelengths of 590 nm (*λ*_ex_) and 630 nm (*λ*_em_).

### 2.7. Antibodies, Immunoblotting, and Microscopic Imaging

For analysis of protein expression, 30 *μ*g of protein isolated from whole-cell lysates and wide-range molecular weight standards (Precision Plus Protein Kaleidoscope, Bio-Rad Polska, Warsaw, Poland) was electrophoresed on a 7.5, 10, or 12% acrylamide SDS-PAGE gels and immunoblotted onto polyvinylidene difluoride Immun-Blot PVDF membranes (Bio-Rad Polska, Warsaw, Poland). The membranes were blocked for 1 h in room temperature either with 5% nonfat dry milk (NFDM) in TBST (NaCl 137 mM, KCl 2.7 mM, and Tris base 19 mM) or in 5% BSA in TBST (depending on the antibody used). Cells were cultured with or without experimental factors indicated in figure legends, harvested, washed, and lysed with RIPA lysis buffer (Santa Cruz Biotechnologies, Santa Cruz, CA, USA) was added. To lyse the cell pellets, cells were broken up by repetitive triturating with the syringe with attached needle (21 G, 0.8 mm diameter). Cell suspension was then left on ice (4°C) for 30 min and centrifuged for another 5 min (4°C, 8,000 ×g). Next, viscous solution was divided into smaller volumes and transferred to fresh Eppendorf tubes and stored at −80°C until being used. For protein quantification in the whole-cell lysates, a protein-dye-binding method [33] with commercial reagent was used (Bio-Rad Laboratories, Hercules, CA, USA).

Antibodies against listed proteins were used: actin, clusterin (Santa Cruz Biotechnologies, Santa Cruz, CA, USA), APP, sAPP*α*, beta amyloid 1-16 (6E10) (Covance, Inc., NY, USA), APP (Merck Millipore, Darmstadt, Germany), MAP LC3 (Novus Biologicals, Cambridge, UK), and VPS34 (Thermo Fisher Scientific, Pierce Biotechnology, IL, USA). Working antibody concentrations (from 1 : 200 to 1 : 2000) varied depending on the protein detected and were applied according to the manufacturer's recommendation. Secondary polyclonal antibodies (Santa Cruz Biotechnology, Santa Cruz, CA, USA) raised against respective species and conjugated to horseradish peroxidase were used for detection, followed by enhanced chemiluminescence assay (Amersham International, Aylesbury, UK). After exposure and processing the films were scanned and analyzed using Kodak EDAS 290/Kodak 1D 3.5 system.

Morphological changes and cell survival were monitored under an inverted phase-contrast and fluorescent microscope, respectively (Olympus CK40, model ICD703WP, and Olympus IX71 Series, Osaka, Japan). The formation of neural cells was monitored by obtaining photographs using digital camera (supplementary material Figure 2, Olympus Camera, Tokyo, Japan).

Demonstration of the presence and intracellular location of certain modifications (autophagosomes, autophagic vacuoles, and multivesicular and multilamellar bodies) was based on ultrastructural studies (transmission electron microscopy (TEM)). Cells were fixed in 2.5% paraformaldehyde and 2% glutaraldehyde in 0.1 M sodium cacodylate buffer (pH 7.4) for 2 h at 4°C. Cells were washed with the same buffer and postfixed with 1% OsO_4_ in 0.1 M sodium cacodylate buffer for 1 h. Next, cells were dehydrated in a graded ethanol alcohol series and embedded in Epon 812. Ultrathin sections were mounted on copper grids, air-dried, and stained for 10 min with 4.7% uranyl acetate and for 2 min with lead citrate. The sections were examined and photographed with a JEOL JEM 1011 electron microscope (Jeol, Tokyo, Japan).

### 2.8. Cellular A*β* 1-40 Assays

Production of A*β* 1-40 was measured in PC-12-transfected cells expressing wild-type human* APP* (*APP-wt*, W), Swedish mutated APP (*APP-sw*, S), empty vector (*GFP* only, G), and complementary DNA (cDNA). Cells were seeded overnight at 3 × 10^4^ cells per well in a 96-multiwell plate. Cells were incubated in DM for 48 h and washed with PBS, and fresh RM media were added for another 48 h with or without experimental factors. Next, cellular media were harvested and assayed for the presence of A*β* 1-40 with an A*β* 1-40 homogenous time resolved fluorescence (HTRF) assay (CisBio) according to manufacturer's instructions. For all cell types (W, S, and G), A*β* 1-40 values were normalized for cell viability, as determined with the MTT assay.

### 2.9. Statistical Analysis

Each experiment was repeated at least three times. The data are expressed as the means ± SEM. Statistical analyses were performed using one-way analysis of variance (ANOVA) followed by Kruskal-Wallis, Tukey's, Newman-Keuls, or Benferroni multiple range test. Regression analysis (linear) was carried out to draw appropriate standard curves. *P* values of less than 0.05 were considered statistically significant. Statistical differences from nontreated control cells were indicated by different lower case letters (*P* < 0.05, bar charts). Statistical analyses were performed using GraphPad Prism version 5.0 software (GraphPad Software Inc., San Diego, CA, USA).

## 3. Results

### 3.1. Viability of Nucleofected PC-12 Cells Is Markedly Reduced by APP (*APP-sw*) but It Is Not Affected by TNF-*α*, TRAIL, Atorvastatin, Simvastatin, Nystatin, or M*β*CD

**Figure 1:**
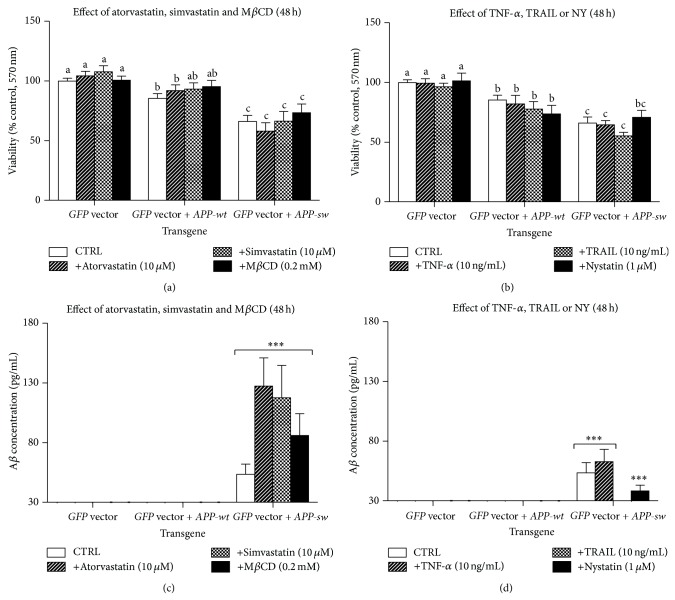
Bar charts (means ± SEM) represent cell viability (MTT assay, upper panel) or A*β* 1-40 concentration in supernatants (HTRF assay, lower panel) normalized for cell viability as determined with the MTT assay measured in the same wells of multiwell plates. Values are expressed as % of control (untreated PC-12 cells nucleofected with* GFP*, or* GFP* +* APP-wt*, or* GFP* +* APP-sw*). ((a)–(c)) The effect of atorvastatin (10 *μ*M), simvastatin (10 *μ*M), or M*β*CD (0.2 mM); ((b)–(d)) the effect of TNF-*α* (10 ng/mL), TRAIL (10 ng/mL), or nystatin (1 *μ*M). Different lower case letters indicate statistically significant differences between means (*P* < 0.05).

Nucleofected PC-12 cells (*GFP* vector,* GFP* vector +* APP-wt,* or* GFP* vector +* APP-sw*) after two days of differentiation in DM were left untreated or treated with TNF-*α* (10 ng/mL), TRAIL (10 ng/mL), atorvastatin (5 *μ*M), simvastatin (5 *μ*M), nystatin (1 *μ*M), or M*β*CD (0.2 *μ*M) for another two days in RM. Cell viability (MTT and NR assays) expressed as percent of control value (untreated cells) was significantly reduced after* APP-sw* cell nucleofection (Figures 1(a) and 1(b), supplementary material Figure 4). No effect of the above-mentioned experimental factors was observed irrespectively to nucleofection (*GFP* vector,* GFP* vector +* APP-wt,* or* GFP* vector +* APP-sw*) (*P* > 0.05). In turn, percentage of YO-PRO-1 and PI-positive cells in relation to nontreated cells (% control) did not differ markedly between the transgene or type of treatment (data not shown). It points to other than apoptosis and necrosis cell death mechanism responsible for lower cell viability after* APP-sw* cell nucleofection.

### 3.2. Production of *β*-Amyloid 1-40 Is Markedly Elevated in* GFP* Vector +* APP-sw* Nucleofected Cells Comparing to* GFP* Vector and* GFP* Vector +* APP-wt*-Nucleofected Cells

The concentration of immunoreactive *β*-amyloid 1-40 (nonfibrillogenic form) in supernatants of* GFP* vector +* APP-sw*-nucleofected cells collected from 96-multiwell plates used to determine cell viability increased significantly to 53.58 pg/mL (Figures 1(c) and 1(d)). No changes in *β*-amyloid 1-40 concentration were found in supernatants collected from remaining untreated cell cultures (*GFP* vector and* GFP* vector +* APP-wt*, *P* > 0.05). Neither administration of TNF-*α*, TRAIL, atorvastatin, simvastatin, nystatin, or M*β*CD led to significant elevation of *β*-amyloid 1-40 concentration in supernatants collected from* GFP* vector +* APP-sw* nucleofected cells as the observed increase was at the cut-off line for HTRF method (according to manufacturer *β*-amyloid 1-40 detected in supernatants at concentration of 30 pg/mL and below has to be neglected Figures 1(c) and 1(d)).

### 3.3. *GFP* Vector +* APP-sw*-Nucleofected PC-12 Cells Show Symptoms of Excessive Formation of Autophagosomes and Multivesicular Bodies but Not Autolysosomes

**Figure 2:**
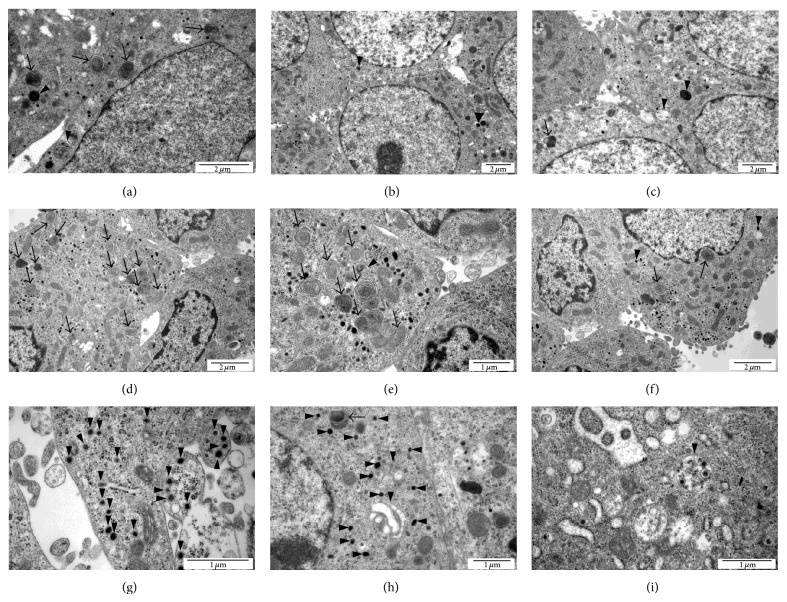
Micrographs (TEM) show ultrastructure of PC-12 cells nucleofected with* GFP* ((a)–(c)), or* GFP* +* APP-wt* ((d)–(f)), or* GFP* +* APP-sw* ((g)–(i)). Symptoms of autophagy: black arrows indicate multilamellar bodies and black arrowheads indicate autophagosomes, multivesicular bodies, and lysosomes. Abnormal autophagy in* GFP* +* APP-sw*-nucleofected cells is manifested by buildup of autophagosomes with double membrane (g) and multivesicular bodies (h); large autophagosome with double membrane contains smaller autophagosomes (i) and there is scarce evidence for single-membrane autolysosomes. Bars represent 100 *μ*m.

Ultrastructural examination of PC-12-nucleofected cells (*GFP* vector,* GFP* vector +* APP-wt*) with TEM revealed little evidence of autophagy. As shown in respective micrographs, the symptoms of autophagy-like process were observed regardless of nucleofection (*GFP* vector,* GFP* vector* + APP-wt,* or* GFP* vector +* APP-sw*); however, the intensity of autophagic phenotype was considerably advanced in* GFP* vector +* APP-sw*-nucleofected PC-12 cells (Figure 2). There is scarce evidence for ER abnormalities in micrographs representing* APP-sw*-nucleofected PC-12 cells (polysomes and reticular tubules are regularly distributed). Nevertheless, as some tubules seem to be distended we suggest that it may represent phagophores in the elongation phase.

### 3.4. Expression of Clusterin Protein Is Not Affected by Nucleofection (*GFP* Vector,* GFP* Vector +* APP-wt*, or* GFP* Vector +* APP-sw*) Although It Is Markedly Increased after TRAIL or Nystatin Administration; Additional Treatment with Rapamycin (1 *μ*M) or Chloroquine (30 *μ*M) for 1 Hour Revealed the Highest Expression of MAP LC3-I, LC3-II, and VPS34 Proteins in* GFP* Vector +* APP-sw*-Nucleofected Cells

**Figure 3:**
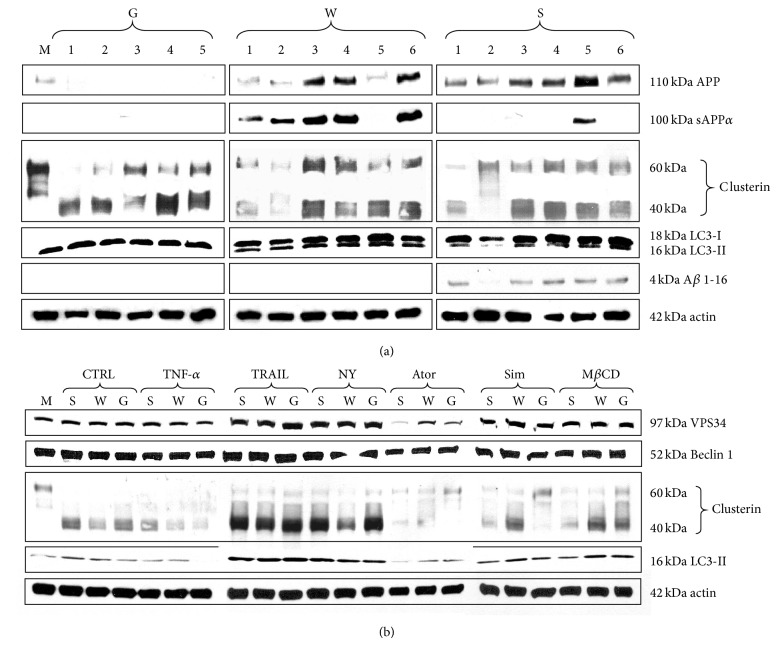
Analysis of protein expression. Letter “M” indicates mock-nucleofected cells, while G, W, S stand for PC-12 cells nucleofected with* GFP* vector (G), or* GFP* vector +* APP-wt* (W), or* GFP* vector +* APP-sw* (S), respectively. (a) Identification of APP, sAPP*α*, clusterin, LC3-II, A*β* 1-16, and beta-actin in whole cell lysates isolated from six subsequent nucleofections; (b) identification of VPS34, Beclin 1, clusterin, LC3-II, and beta-actin in whole cell lysates isolated from cells nucleofected with* GFP* vector (G), or* GFP* vector +* APP-wt* (W), or* GFP* vector +* APP-sw* (S).

Immunoblotting carried out with PVDF membranes loaded with proteins separated by SDS-PAGE from six subsequent nucleofections (1-6) unraveled that* APP* genes had no significant effect on the expression of secretory clusterin (sCLU, Figure 3(a)). Expression of clusterin protein rose, however, after treatment with TRAIL or nystatin (Figure 3(b)). The sAPP*α* protein (*α*-secretase product) was exclusively observed in* GFP* vector +* APP-wt*-nucleofected cells, except lane 5 representing mixture of* GFP* vector +* APP-wt* plus* GFP* vector +* APP-sw*-nucleofected cells (positive control) (Figure 3(a)). In turn, total APP protein expression (unprocessed and processed form) was exclusively detected in* APP* gene-nucleofected cells (*GFP* vector +* APP-wt* and* GFP* vector +* APP-sw*, Figure 3(a)). In contrast, the expression of immunoreactive *β* amyloid 1-16 (any form) was absent in* GFP* vector and* GFP* vector +* APP-wt* and found solely in* GFP* vector +* APP-sw*-nucleofected cells (Figure 3(a)). Markers of autophagy (VPS34, MAP LC3-II, and Beclin 1) were also monitored with WB. Functionally active MAP LC3-II (LC3-II) was detected in* GFP* vector,* GFP* vector +* APP-wt*-, and* GFP* vector +* APP-sw*-nucleofected cells (Figure 3(a)). Interestingly, expressions of early autophagy marker VPS34 (class III phosphoinositide 3-kinase, PI3K III) and LC3-II were significantly elevated after TRAIL, nystatin, simvastatin, or M*β*CD administration (Figure 3(b)). TNF-*α* and atorvastatin apparently diminished clusterin and LC3-II expressions, even though they did not affect VPS34 expression levels (Figure 3(b)). No effect of treatment was found in the expression of Beclin 1, protein important at the sequestration stage of autophagy (Figure 3(b)). To make the issue of autophagy clearer, additional “flux” experiment was performed. After the experiment ended, the cells were additionally treated with rapamycin (1 *μ*M) or chloroquine (30 *μ*M) for 1 hour. As it is presented in supplementary material Figure 5, the highest expression of LC3-I, LC3-II, and VPS34 proteins was observed in* GFP* vector +* APP-sw*-nucleofected cells.

## 4. Discussion

Incompetent autophagy causes decline of cell viability resulting from accumulation of nonfunctional organelles, proteins, and protein aggregates. Progressive changes inevitably end up in cell death but the precise link mechanisms and type of cell death beneath are unknown. We could not demonstrate that reduced viability of nucleofected cells was caused by apoptotic and/or necrotic cell death as percent of YO-PRO-1 and PI-positive cells did not differ significantly irrespectively of transgene or treatment (data not shown). Discussion about any other types of cell death (necroptosis and/or paraptosis) that could account for drop in percentage of viable cells in this experiment would be highly speculative. The paradox of autophagy is that it is essential for keeping cellular homeostasis, and consequently any disruption of this homeostasis results in severe effects [34]. Autophagy impairment as a method of tumor cell elimination provides strong rationale for developing strategies other than apoptosis induction. Lysosomal membrane permeabilization (LMP) which is known to occur in lysosome storage diseases (LSD) represents large group of disturbances where autophagy went wrong. LMP is frequently observed in AD where it is associated with elevated lysosomal pH [35]. Definitely, nucleofection of PC-12 with mutated* APP* gene was confirmed at genomic (PCR) and translational levels (WB) (Figure 3(a), supplementary material Figure 3). It led to significant drop of cell viability (by 36%) accompanied by prevalent autophagy-like process. Possible ultrastructural modifications of ER including excessive formation of phagophores might result from the expression of mutated form of APP and impaired lysosome fusion with autophagosomes. Why the* APP-sw*-nucleofected PC-12 cells have nonfunctional modifications in cellular organelle essential for autophagy is not known, although numerous papers emphasize the importance of this process in pathogenesis of Alzheimer's disease [19, 20]. Moreover, the autophagy could not reverse lower cell viability suggesting that it could go awry. Cautious analysis of micrographs obtained in TEM provided evidence of extensive formation of autophagosomes as well as numerous multivesicular bodies with little presence of autolysosomes in* APP-sw*-nucleofected PC-12 cells (Figures 2(g)–2(i)). Neither experimental factor affected cell viability confirming extreme resistance of PC-12 cells to death receptor ligands (TNF-*α*, TRAIL) or cholesterol inhibitors (atorvastatin, simvastatin, nystatin, and M*β*CD), (Figure 1(a)). Previously, statins were reported to induce autophagy and inhibit viability of cancer cells [36–38]. Apparently, in our study lower cell viability had predominantly something to do with changes induced by the expression of* APP-sw* gene (*P* < 0.001 by two-way analysis of variance). Actually, significant rise of human A*β* 1-40 secretion was solely noticed in* APP-sw*-nucleofected PC-12 cells (from undetectable levels to 53.83 pg/mL, *P* < 0.05). Elevated concentration of A*β* in supernatants collected from* APP-sw*-nucleofected PC-12 cell cultures was substantiated by the results of WB. The expression of A*β* 1-16 peptide (any form: A*β* 1-40, A*β* 1-42, and A*β* 1-43) was detected in* APP-sw*-nucleofected but not in* GFP* vector- or* GFP* vector +* APP-wt*-nucleofected cell cultures (Figure 3(a)). It is important to stress that each form of A*β* evokes distinct effects in affected cells. In general, AD pathology and neuronal death are associated with excessive production of A*β*, but mainly A*β* 1-42 peptide is severely fibrillogenic (the source of fibrilles and senile plaques). In turn, the expression of sAPP*α*, the protein product of *α*-secretase which rules out the formation of A*β*, was found entirely in* GFP* vector +* APP-wt*-nucleofected cell cultures (Figure 3(a)). The latter observation authenticates APP695 as highly specific substrate for *α*-secretase activity. Finally, total APP expression levels (both processed and unprocessed form) measured by immunoblotting showed this protein in* GFP* vector +* APP-wt*- and* GFP* vector +* APP-sw*-nucleofected cells only (Figure 3(a)). These results are consistent with a common view addressing importance of A*β* in etiology and pathogenesis of AD and also advocate assumption that A*β* is harmful to PC-12 cells. How does A*β* affect autophagy is not known at present and needs additional scrutiny. Expression of clusterin (CLU) protein which represents unique ATP-independent extracellular chaperone was measured to find out if this protein is affected by nucleofection, treatment, and autophagy. No changes were detected irrespectively to type of nucleofection in six subsequent nucleofections (Figure 3(a)). The expression of protein markers of initial steps of autophagy (VPS34 and LC3-I, LC3-II, and Beclin 1) did not differ between transgenes or the type of treatment (Figures 3(a) and 3(b)) but the image markedly changed after final 1-hour treatment with rapamycin (1 mM, RAPA) or chloroquine (30 mM, CHLOR). This “flux” experiment demonstrated that autophagy was incomplete and has the highest rate in the* GFP* vector +* APP-sw*-nucleofected cells as autophagy inhibitor at the level of autolysosome formation (CHLOR) led to the accumulation of LC3-I/ LC3-II (supplementary material Figure 5). Additionally, autophagy stimulator rapamycin was unable to reverse the effect of chloroquine. Thus, it is essential to monitor autophagy with including stimulators/inhibitors (RAPA, CHLOR). The use of inhibitor (CHLOR) revealed that lack of differences in the expression of VPS34 and LC3-I, LC3-II, and Beclin 1 between the* GFP* vector and* GFP* vector +* APP-wt*-nucleofected cells was probably due to lysosome-dependent degradation. As mentioned earlier the LC3-I is activated by APG7L/ATG7, transferred to ATG3, and conjugated to phospholipid (PE) to form LC3-II. It should be stressed that soluble form of LC3-II is not observed in PC-12 cells and thus LC3-II bands shown in the respective immunoblots represent LC3-II anchored to phagophores. However, similarly to VPS34 and LC3-II, CLU expression levels increased in response to TRAIL or nystatin treatment pointing to possible involvement of this protein in early phase of autophagy. Given that TRAIL or nystatin administration was able to increase expression of incompletely glycosylated 40 and 60 kDa CLU variants (Figure 3(b)) in either type of transgene bearing cells, it remains unclear whether this effect is directly linked to autophagy. It is not possible that clusterin simply is induced by TRAIL and NY in all types of cells and has nothing to do, at least here, with autophagy. As far as we know, this is first report showing that VPS34, LC3-II, and CLU are elevated in parallel when PC-12 cells are challenged with TRAIL or nystatin. No obvious link between these proteins and clusterin was demonstrated so far.

## 5. Conclusions

Pheochromocytoma PC-12 cells are completely resistant to treatment with death receptor ligands (even two days with 100 ng/mL of TNF-*α* or TRAIL combined with 1 nM of actinomycin D had no effect on cell viability, data not shown). Nonetheless, cell viability dropped significantly in* GFP* vector +* APP-sw*-nucleofected cells but not in* GFP* vector- or* GFP* vector +* APP-wt*-nucleofected cells. At the same time just* GFP* vector +* APP-sw*-nucleofected cells expressed A*β* 1-16 and secreted A*β* 1-40. At the same time profound autophagy-like process occurred with numerous autophagosomes and multivesicular bodies but with scarce evidence of autolysosomes. Summing up, human* APP-sw* gene is apparently destructive to PC-12 cells as cells are driven to incomplete autophagy-like process. It seems that CLU protein accompanies early phase of autophagy (isolation and sequestration).

## Supplementary Material

Figure S1: The Vector Map of pCMV6-AC-GFP.
Figure S2: Phase-contrast and fluorescent views showing the phenotype of *GFP* vector (G), or *GFP* vector + *APP-wt* (W), or *GFP* vector + *APP-sw* (S) nucleofected PC-12 cells (24 hours after nucleofection). Bars represent 100 *μ*m.
Figure S3: Analysis of PCR products.
Figure S4: Bar charts (means + SEM) represent cell viability (NR assay) expressed as % of control (untreated PC-12 cells nucleofected with *GFP*, or *GFP* + *APP-wt*, or *GFP* + *APP-sw*). Different lower case letters indicate statistically significant differences between means (*P* < 0.05).
Figure S5: Analysis of protein expression in the “flux” experiment additionally treated with rapamycin (1 *μ*M) or chloroquine (30 *μ*M) for the last hour of experiment.
