# Supplementary figures and images for: Nucleofection of Rat Pheochromocytoma PC-12 Cells with Human Mutated Beta-Amyloid Precursor Protein Gene (APP-sw) Leads to Reduced Viability, Autophagy-Like Process, and Increased Expression and Secretion of Beta Amyloid
Source: Biomed Res Int. 2015 Mar 10;2015:746092. doi: 10.1155/2015/746092 (PMC4363875; doi:10.1155/2015/746092)

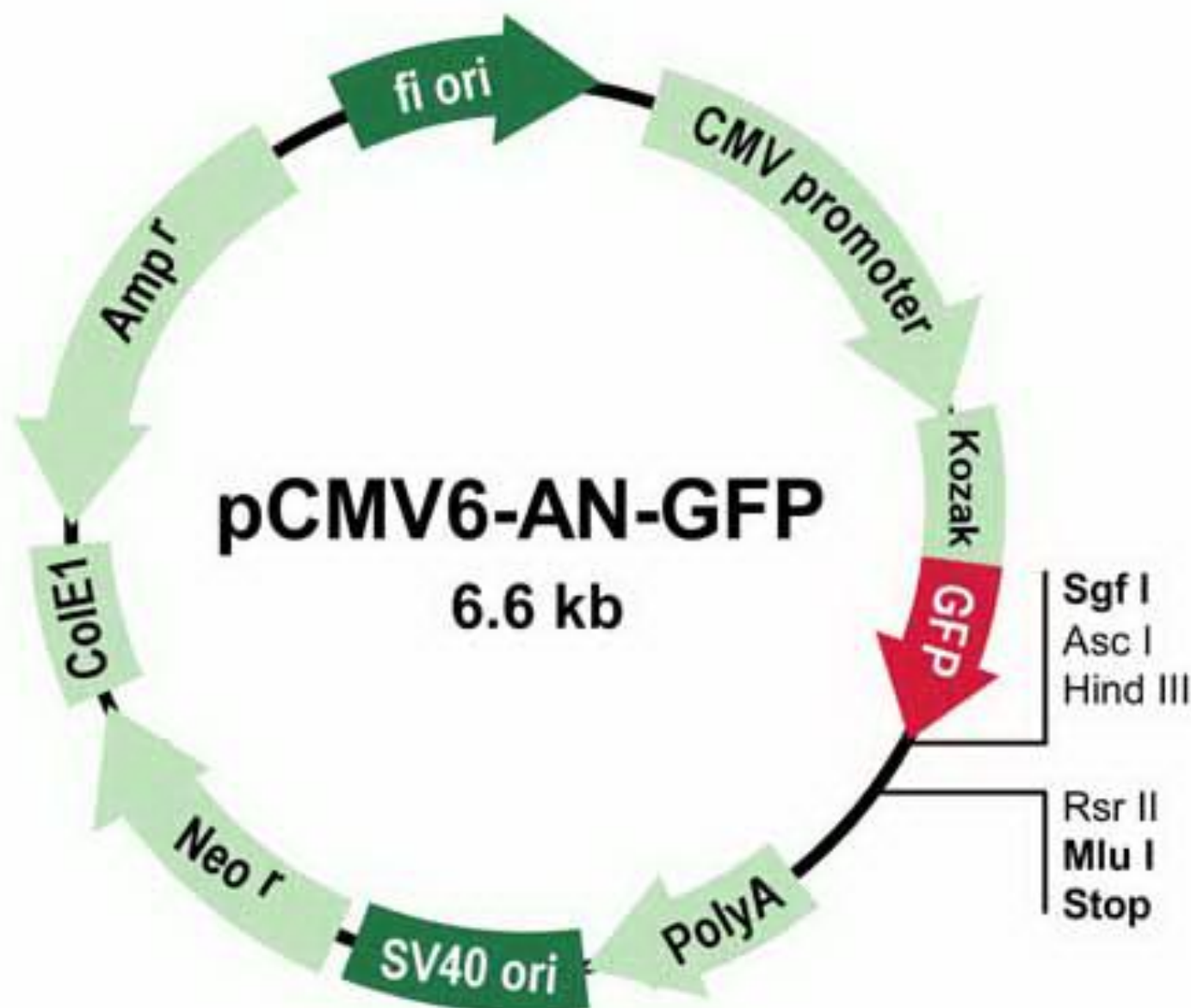

Supplement: Supplementary file 1 — Figure S1: The Vector Map of pCMV6-AC-GFP. Figure S2: Phase-contrast and fluorescent views showing the phenotype of GFP vector (G), or GFP vector + APP-wt (W), or GFP vector + APP-sw (S) nucleofected PC-12 cells (24 hours after nucleofection). Bars represent 100 μm. Figure S3: Analysis of PCR products. Figure S4: Bar charts (means + SEM) represent cell viability (NR assay) expressed as % of control (untreated PC-12 cells nucleofected with GFP, or GFP + APP-wt, or GFP + APP-sw). Different lower case letters indicate statistically significant differences between means (P < 0.05). Figure S5: Analysis of protein expression in the “flux” experiment additionally treated with rapamycin (1 μM) or chloroquine (30 μM) for the last hour of experiment. [file 746092.f1.zip › Figure S1.pdf]

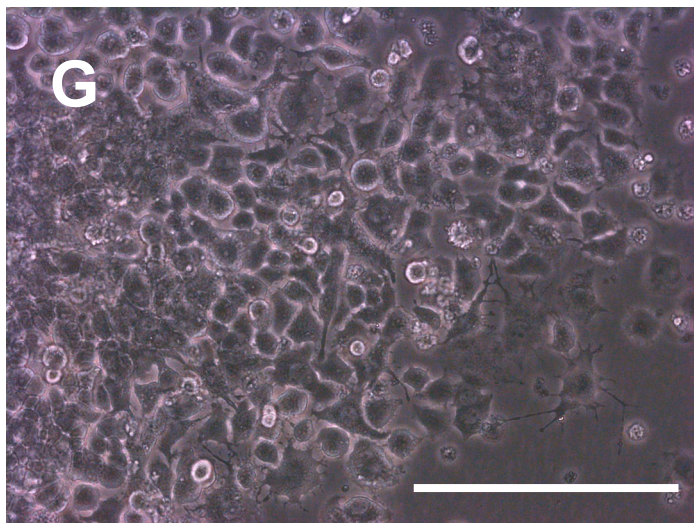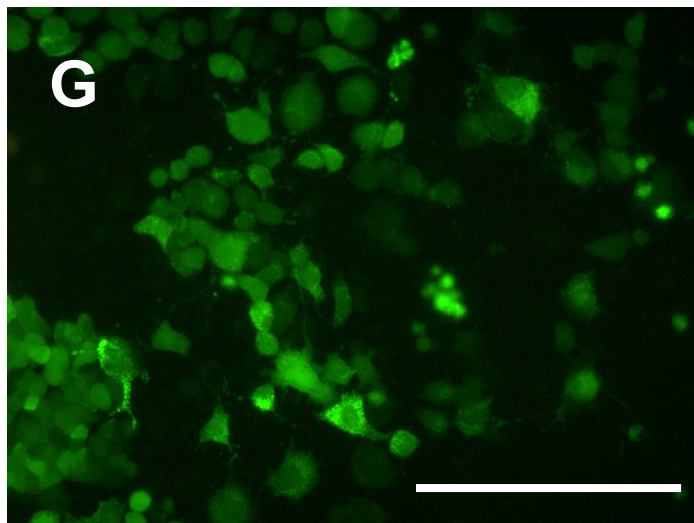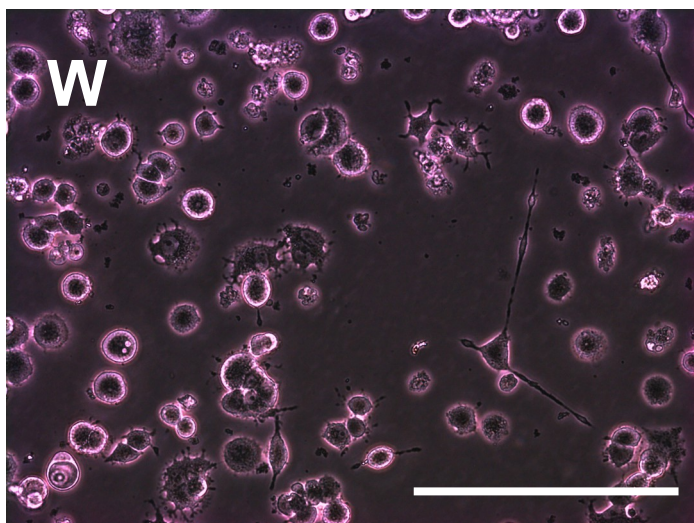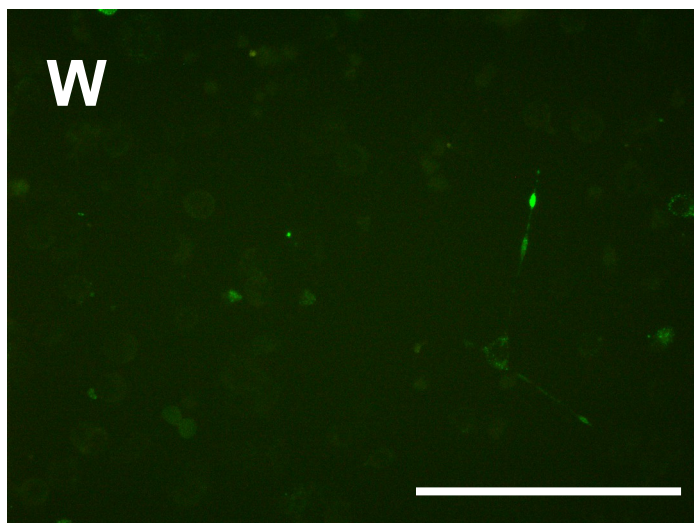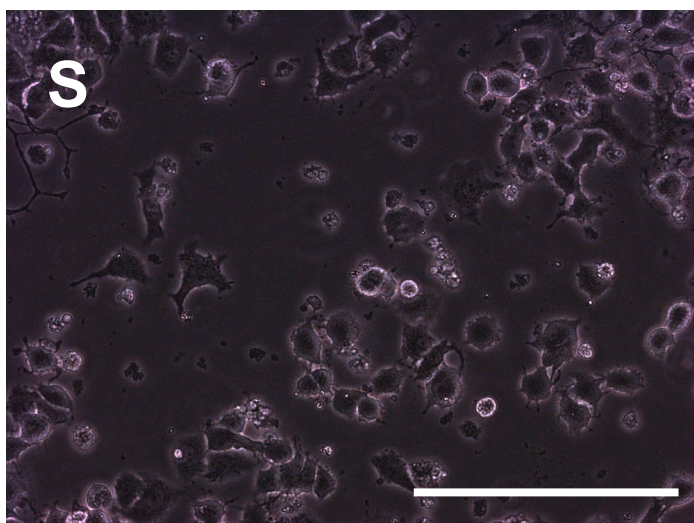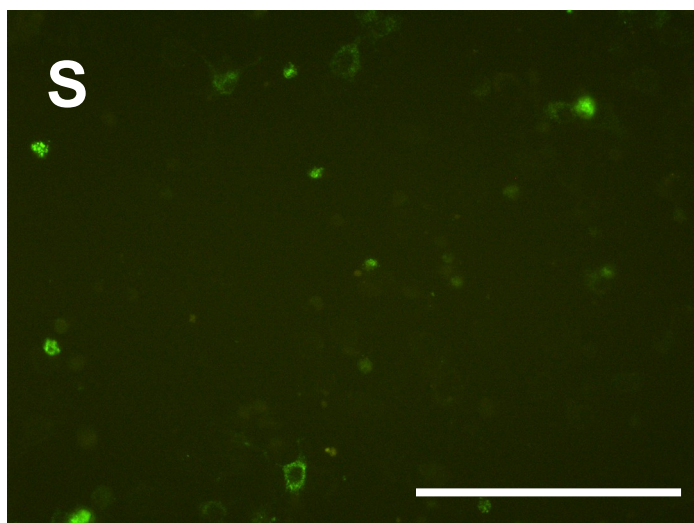

Supplement: Supplementary file 1 — Figure S1: The Vector Map of pCMV6-AC-GFP. Figure S2: Phase-contrast and fluorescent views showing the phenotype of GFP vector (G), or GFP vector + APP-wt (W), or GFP vector + APP-sw (S) nucleofected PC-12 cells (24 hours after nucleofection). Bars represent 100 μm. Figure S3: Analysis of PCR products. Figure S4: Bar charts (means + SEM) represent cell viability (NR assay) expressed as % of control (untreated PC-12 cells nucleofected with GFP, or GFP + APP-wt, or GFP + APP-sw). Different lower case letters indicate statistically significant differences between means (P < 0.05). Figure S5: Analysis of protein expression in the “flux” experiment additionally treated with rapamycin (1 μM) or chloroquine (30 μM) for the last hour of experiment. [file 746092.f1.zip › Figure S2.pdf]

A

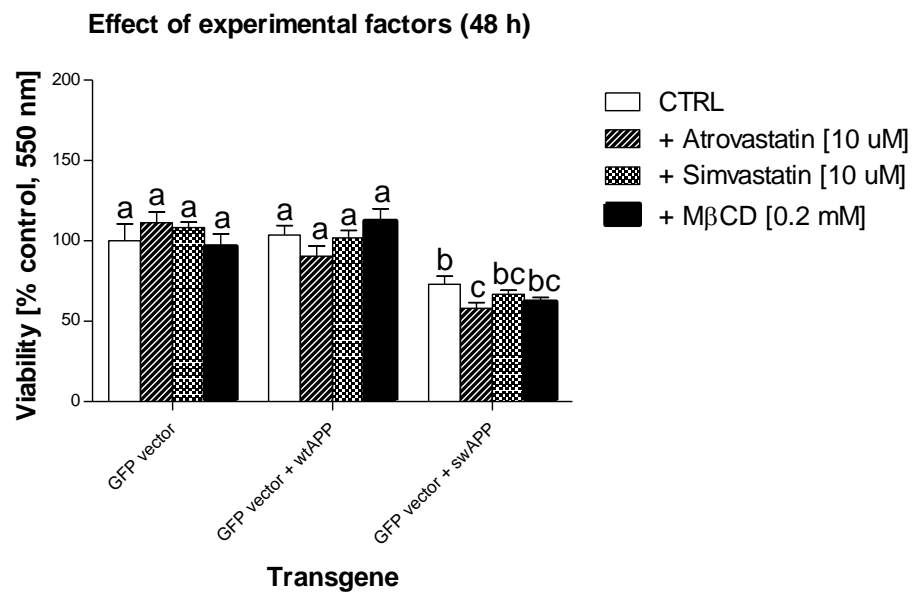

B

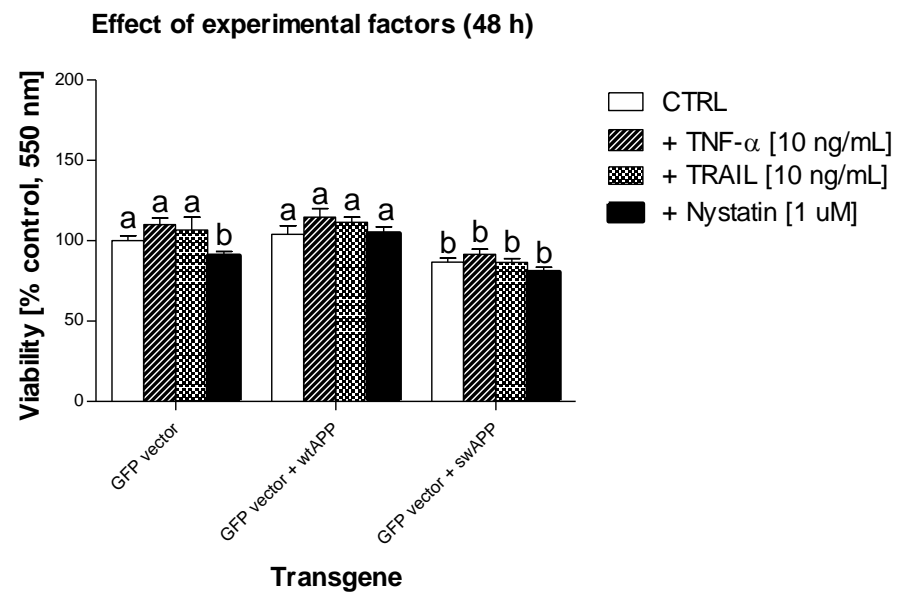

Supplement: Supplementary file 1 — Figure S1: The Vector Map of pCMV6-AC-GFP. Figure S2: Phase-contrast and fluorescent views showing the phenotype of GFP vector (G), or GFP vector + APP-wt (W), or GFP vector + APP-sw (S) nucleofected PC-12 cells (24 hours after nucleofection). Bars represent 100 μm. Figure S3: Analysis of PCR products. Figure S4: Bar charts (means + SEM) represent cell viability (NR assay) expressed as % of control (untreated PC-12 cells nucleofected with GFP, or GFP + APP-wt, or GFP + APP-sw). Different lower case letters indicate statistically significant differences between means (P < 0.05). Figure S5: Analysis of protein expression in the “flux” experiment additionally treated with rapamycin (1 μM) or chloroquine (30 μM) for the last hour of experiment. [file 746092.f1.zip › Figure S4.pdf]

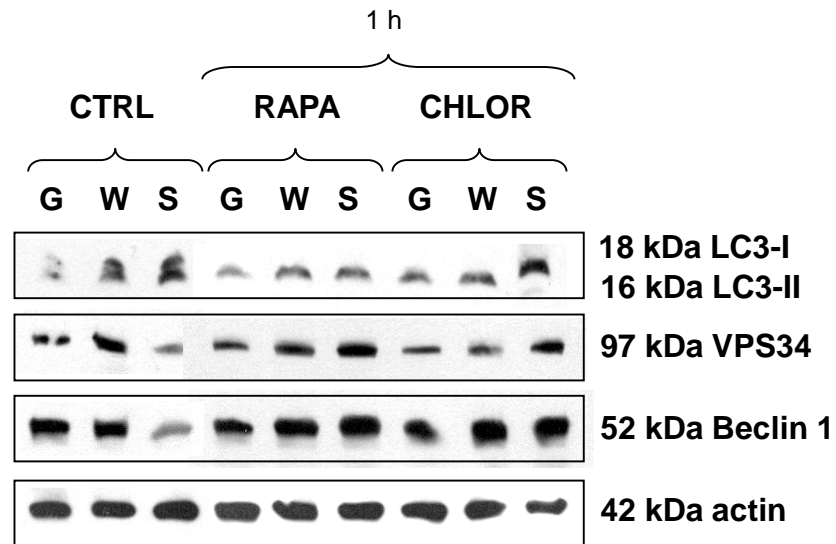

Supplement: Supplementary file 1 — Figure S1: The Vector Map of pCMV6-AC-GFP. Figure S2: Phase-contrast and fluorescent views showing the phenotype of GFP vector (G), or GFP vector + APP-wt (W), or GFP vector + APP-sw (S) nucleofected PC-12 cells (24 hours after nucleofection). Bars represent 100 μm. Figure S3: Analysis of PCR products. Figure S4: Bar charts (means + SEM) represent cell viability (NR assay) expressed as % of control (untreated PC-12 cells nucleofected with GFP, or GFP + APP-wt, or GFP + APP-sw). Different lower case letters indicate statistically significant differences between means (P < 0.05). Figure S5: Analysis of protein expression in the “flux” experiment additionally treated with rapamycin (1 μM) or chloroquine (30 μM) for the last hour of experiment. [file 746092.f1.zip › Figure S5.pdf]
